# Supplementary material for: Structures of diverse poxin cGAMP nucleases reveal a widespread role for cGAS-STING evasion in host–pathogen conflict
Source: eLife. 2020 Nov 16;9:e59753. doi: 10.7554/eLife.59753 (PMC7688311; doi:10.7554/eLife.59753)
Supplement: Supplementary file 2. — This file summarizes data collection, phasing, and refinement statistics for X-ray crystallography data. All crystallography datasets were collected from individual crystals. Values in parentheses are for the highest resolution shell. [file elife-59753-supp2.docx]

**Supplementary File 2: Crystallographic statistics, Related to Figures 2–4 and 6**

|  | **AcNPV poxin**  **Post-reactive state** | **PrGV poxin**  **Post-reactive state** | ***Trichoplusia ni* poxin**  **Post-reactive state** | ***Danaus plexippus* poxin**  ***Apo*** |
| --- | --- | --- | --- | --- |
| **Data Collection** | | |  |  |
| Resolution (Å)^a^ | 38.31–1.90 (1.93–1.90) | 38.64–1.90 (1.94–1.90) | 39.57–2.89 (3.07–2.89) | 38.12–1.45 (1.47–1.45) |
| Wavelength (Å) | 0.97930 | 0.97918 | 0.99995 | 0.97890 |
| Space group | P 1 | P 1 2_1_ 1 | I 4_1_ | P 1 2_1_ 1 |
| Unit cell: a, b, c (Å) | 127.23, 127.19, 127.15 | 77.47, 58.14, 100.70 | 124.42, 124.42, 132.18 | 44.16, 75.41, 77.42 |
| Unit cell: α, β, γ (°) | 124.81, 102.39, 102.39 | 90.00, 108.55, 90.00 | 90.0, 90.0, 90.0 | 90.00 100.06, 90.00 |
| Molecules per ASU | 16 | 4 | 2 | 2 |
| Total reflections | 1,582,102 | 468,797 | 341,829 | 297,811 |
| Unique reflections | 422,103 | 66,856 | 22,422 | 87,244 |
| Completeness (%)^a^ | 92.3% (90.0%) | 99.4 (95.7) | 99.6 (97.9) | 98.6 (94.9) |
| Multiplicity^a^ | 3.7 (3.6) | 7.0 (6.1) | 15.2 (14.7) | 3.4 (3.2) |
| *I/σI^a^* | 10.5 (1.3) | 9.7 (1.4) | 9.5 (1.1) | 7.2 (1.3) |
| CC(1/2)^b^ (%)^a^ | 0.994 (0.623) | 0.997 (0.472) | 0.996 (0.372) | 0.996 (0.441) |
| Rpim^c^ (%)^a^ | 4.5 (58.1) | 5.7 (93.4) | 6.8 (85.4) | 5.1 (71.5) |
| Sites |  |  |  |  |
| **Refinement** | | |  |  |
| Resolution (Å) | 38.31–1.90 | 38.64–1.90 | 39.57–2.90 | 38.13–1.45 |
| Free reflections | 2037 | 2008 | 2009 | 4203 |
| R-factor / R-free | 17.48 / 20.83 | 18.26 / 20.68 | 18.41 / 21.46 | 15.69 / 17.43 |
| Bond distance (RMS Å) | 0.007 | 0.002 | 0.006 | 0.013 |
| Bond angles (RMS °) | 1.013 | 0.568 | 0.934 | 1.174 |
| Twin Law | -l, -h, h+k+l |  |  |  |
| **Structure/Stereochemistry** | | |  |  |
| No. atoms: protein | 29,078 | 6216 | 3726 | 3834 |
| No. atoms: ligand | 736 | 138 | 92 |  |
| No. atoms: solvent | 3,334 | 749 |  | 640 |
| Average B-factor: protein | 27.54 | 34.79 | 75.35 | 19.01 |
| Average B-factor: ligand | 24.05 | 41.08 | 79.86 |  |
| Average B-factor: solvent | 35.77 | 38.64 |  | 29.95 |
| Ramachandran plot: favored | 97.62% | 98.00% | 96.43% | 98.73% |
| Ramachandran plot: allowed | 2.38% | 1.73% | 3.36% | 1.27% |
| Ramachandran plot: outliers | 0.00% | 0.27% | 0.21% | 0.00% |
| Rotamer outliers | 1.95% | 0.00% | 0.50% | 0.24% |
| MolProbity^d^ score | 1.96 | 1.05 | 1.54 | 1.09 |
| Protein Data Bank ID | 6XB3 | 6XB4 | 6XB5 | 6XB6 |

^a^ Highest resolution shell values in parentheses

^b^ (Karplus and Diederichs, 2012)

^c^ (Weiss, 2001)

^d^ (Chen et al., 2010)

**Supplementary Table 2 (Cont.): Crystallographic Statistics, Related to Figures 2–4 and 6**

|  | **AcNPV Poxin**  **(Se-SAD)** | **PrGV Poxin**  **(Se-SAD)** | ***Danaus plexippus* Poxin**  **(Se-SAD)** |
| --- | --- | --- | --- |
| **Data Collection** | | |  |
| Resolution (Å)^a^ | 38.23–2.3 (2.36–2.3) | 38.76–2.3 (2.38–2.30) | 38.17–1.90 (1.94–1.90) |
| Wavelength (Å) | 0.97930 | 0.97918 | 0.97918 |
| Space group | I 4_1_ | P 1 2_1_ 1 | P 1 2_1_ 1 |
| Unit cell: a, b, c (Å) | 159.47, 159.47, 117.26 | 77.67, 57.90, 101.27 | 44.44, 75.74, 82.38 |
| Unit cell: α, β, γ (°) | 90.00, 90.00, 90.00 | 90.00, 108.88, 90.00 | 90.00, 96.13, 90.00 |
| Molecules per ASU | 4 | 4 | 2 |
| Total reflections | 7,136,159 | 790,899 | 601,370 |
| Unique reflections | 64,944 | 38,195 | 42,614 |
| Completeness (%)^a^ | 99.9 (98.4) | 99.9 (99.1) | 99.3 (96.0) |
| Multiplicity^a^ | 109.9 (109.0) | 20.7 (17.3) | 14.1 (13.6) |
| *I/σI^a^* | 29.1 (8.2) | 16.9 (5.3) | 10.1 (2.1) |
| CC(1/2)^b^ (%)^a^ | 0.999 (0.970) | 0.998 (0.957) | 0.998 (0.775) |
| Rpim^c^ (%)^a^ | 2.1 (10.5) | 4.5 (28.3) | 3.5 (37.3) |
| Sites | 20 | 8 | 10 |
| **Refinement** | | |  |
| Resolution (Å) |  |  |  |
| Free reflections |  |  |  |
| R-factor / R-free |  |  |  |
| Bond distance (RMS Å) |  |  |  |
| Bond angles (RMS °) |  |  |  |
| **Structure/Stereochemistry** | | |  |
| No. atoms: protein |  |  |  |
| No. atoms: ligand |  |  |  |
| No. atoms: solvent |  |  |  |
| Average B-factor: protein |  |  |  |
| Average B-factor: ligand |  |  |  |
| Average B-factor: solvent |  |  |  |
| Ramachandran plot: favored |  |  |  |
| Ramachandran plot: allowed |  |  |  |
| Ramachandran plot: outliers |  |  |  |
| Rotamer outliers |  |  |  |
| MolProbity^d^ score |  |  |  |
| Protein Data Bank ID |  |  |  |

^a^ Highest resolution shell values in parentheses

^b^ (Karplus and Diederichs, 2012)

^c^ (Weiss, 2001)

^d^ (Chen et al., 2010)
